# Comparison of Point-of-Care Testing and Hospital-Based Methods in Screening for Potential Type 2 Diabetes Mellitus and Abnormal Glucose Regulation in a Dental Setting

**DOI:** 10.3390/ijerph18126459

**Published:** 2021-06-15

**Authors:** Muneedej Suwattipong, Thitima Thuramonwong, Chanita Tantipoj, Pornpoj Fuangtharnthip, Supanee Thanakun, Weerapan Khovidhunkit, Siribang-on Piboonniyom Khovidhunkit

**Affiliations:** 1 Dental Hospital, Faculty of Dentistry, Mahidol University, Bangkok 10400, Thailand; muneedej.suw@mahidol.edu (M.S.); katae_katier@hotmail.com (T.T.); 2 Department of Advanced General Dentistry, Faculty of Dentistry, Mahidol University, Bangkok 10400, Thailand; ctantipoj@gmail.com (C.T.); pornpoj.fun@mahidol.ac.th (P.F.); 3 College of Dental Medicine, Rangsit University, Muang Pathum Thani 12000, Thailand; supanee.tha2@gmail.com; 4 Department of Medicine, Faculty of Medicine, Chulalongkorn University, Bangkok 10330, Thailand; wkhovid@gmail.com

**Keywords:** point-of-care testing, diabetes mellitus, prevalence, dental clinics, hyperglycemia, abnormal glucose regulation

## Abstract

This study aimed to compare the screening methods between point-of-care (POC) testing and hospital-based methods for potential type 2 DM and abnormal glucose regulation (AGR) in a dental setting. A total of 274 consecutive subjects who attended the Faculty of Dentistry, Mahidol University, Bangkok, Thailand, were selected. Demographic data were collected. HbA_1c_ was assessed using a finger prick blood sample and analyzed with a point-of-care (POC) testing machine (DCA Vantage^®^). Hyperglycemia was defined as POC HbA_1c_ ≥ 5.7%. Random blood glucose (RBG) was also evaluated using a glucometer (OneTouch^®^ SelectSimple™) and hyperglycemia was defined as RBG ≥ 110 mg/dl or ≥140 mg/dl. The subjects were then sent for laboratory measurements for fasting plasma glucose (FPG) and HbA_1c_. The prevalence of AGR (defined as FPG ≥ 100 mg/dl or laboratory HbA_1c_ ≥ 5.7%) and potential type 2 DM (defined as FPG ≥ 126 mg/dl or laboratory HbA_1c_ ≥ 6.5%) among subjects was calculated and receiver operating characteristic (ROC) analysis was performed using FPG and HbA_1c_ for the diagnosis of AGR and potential type 2 DM. The prevalence of hyperglycemia defined as POC HbA_1c_ ≥ 5.7%, RBG ≥ 110 mg/dl, and RBG ≥ 140 mg/dl was 49%, 63%, and 32%, respectively. After the evaluation using laboratory measurements, the prevalence of AGR was 25% and 17% using laboratory FPG and HbA_1c_ criteria, respectively. Based on the ROC curves, the performances of POC HbA_1c_ and RBG in predicting FPG-defined potential type 2 DM were high (AUC = 0.99; 95% CI 0.98–0.99 and AUC = 0.94; 95% CI 0.86–1.0, respectively) but lower in predicting AGR (AUC = 0.72; 95% CI 0.67–0.78 and AUC = 0.65; 95% CI 0.59–0.70, respectively). This study suggested that POC testing might be a potential tool for screening of subjects with potential type 2 DM in a dental setting.

## 1. Introduction

Diabetes mellitus (DM) is a metabolic disease characterized by chronic hyperglycemia resulting from defects in insulin-producing cells, insulin action, or both [1]. The number of people aged ≥20 years estimated to have type 2 DM globally is predicted to increase from 171 million in 2000 to 366 million by 2030 [2]. Undiagnosed type 2 DM are major problems encountered all over the world, and microvascular and macrovascular complications can possibly exist even in patients with prediabetes who had chronic hyperglycemia without any symptoms [2]. According to the data from the Thai National Health Examination Survey 2004, 2009, and 2014, the age-adjusted prevalence of DM reported in Thailand increased from 7.7% in 2004 to 7.8% in 2009 and 9.9% in 2014 (8.9% among men and 10.8% among women). In addition, the proportion of undiagnosed DM remained high in 2014 (51.2% for men and 41.3% for women) [3]. These facts support the urgent need to identify undiagnosed hyperglycemia and type 2 DM earlier.

For decades, the diagnosis of DM has been based primarily on plasma glucose criteria, i.e., measurements of fasting plasma glucose (FPG) and plasma glucose after an oral glucose tolerance test (OGTT). Using American Diabetes Association (ADA) criteria, FPG level of less than 100 mg/dl is classified as normal, between 100–125 mg/dl is classified as prediabetes, and more than or equal to 126 mg/dl is classified as DM [4]. Despite being the diagnostic gold standard for DM, they are more time-consuming, labor-intensive, and impractical for DM screening since these gold standard methods need the patients to fast and cannot be performed after eating. After the discovery of glycated hemoglobin (HbA_1c_), numerous studies have shown that HbA_1c_ could be used as an objective measurement of glycemic control [5]. In 2011, the World Health Organization (WHO) concluded that HbA_1c_ could be used as a diagnostic test for DM in accordance with strict quality assurance and test standardization [6]. According to the ADA, a HbA_1c_ level of less than 5.7% is considered to be normal, a HbA_1c_ between 5.7% and 6.4% is considered to be prediabetes, and a HbA_1c_ level greater than or equal to 6.5% is considered to be DM [4].

In addition to the hospital-based laboratory measurement of HbA_1c_, point-of-care (POC) HbA_1c_ testing has also been used for screening of undiagnosed type 2 DM in many healthcare settings. The use of POC HbA_1c_ as a screening or diagnostic tool has been reported in several studies [7,8,9]. For example, a population-based study conducted in 795 subjects aged 36–60 years in a rural area in Uganda revealed that using POC HbA_1c_, 11.3% of subjects had DM compared with 4.8% for FPG [9]. With FPG as the reference, agreement between FPG and HbA_1c_ in classifying DM status was moderate (Kappa = 22.9; Area Under the Curve (AUC = 75%), while that for abnormal glucose regulation (AGR) was low (Kappa = 11.0; AUC = 59%). However, agreement was high (over 90%) among negative tests and among subjects with risk factors for type 2 DM, including obesity, overweight, and hypertension.

A random blood glucose (RBG) test is also used to screen and diagnose DM when hyperglycemic symptoms are present, along with the RBG level of 200 mg/dl or higher [1]. At present, blood glucose measurement using glucometers as a POC testing has been accepted worldwide for self-monitoring at home as well as for glucose monitoring in hospitalized patients [10].

Several studies have demonstrated that a dental setting could be a good venue for the diagnosis of people with undiagnosed hyperglycemia [11,12,13]. A systematic review regarding the screening for hyperglycemia in dental primary care practice settings was conducted [11]. High rates of undiagnosed hyperglycemia were detected among dental patients using POC testings. In our previous study, dental patients without a history of hyperglycemia were recruited and HbA_1c_ level was assessed using a finger prick blood sample and analyzed with the POC DCA Vantage^®^ analyzer [12]. Of those 724 subjects, 33.8% had hyperglycemia defined as a POC HbA_1c_ level ≥ 5.7%. In this 33.8%, 28.2% had prediabetes defined as a POC HbA_1c_ level of 5.7–6.4% and 5.6% had potential type 2 DM defined as a POC HbA_1c_ level of ≥6.5%. This prevalence was relatively high compared to the data from the NHES IV conducted among 18,629 Thai adults aged ≥20 years, which found that the prevalence of impaired fasting glucose (IFG), defined as having an FPG level from 100–125 mg/dl, and undiagnosed DM, defined as having an FPG level ≥ 126 mg/dl, was 10.6% and 2.3%, respectively [14].

Since there was no report of using POC HbA_1c_ and RBG for diagnosis and screening of potential type 2 DM and AGR in Thailand, this study was conducted to investigate the use of POC HbA_1c_ and RBG in conjunction with symptoms of type 2 DM to screen dental patients for AGR and type 2 DM and to compare the accuracy of these POC measurements with the standardized hospital-based laboratory methods.

## 2. Materials and Methods

### 2.1. Study Population

**Figure 1:**
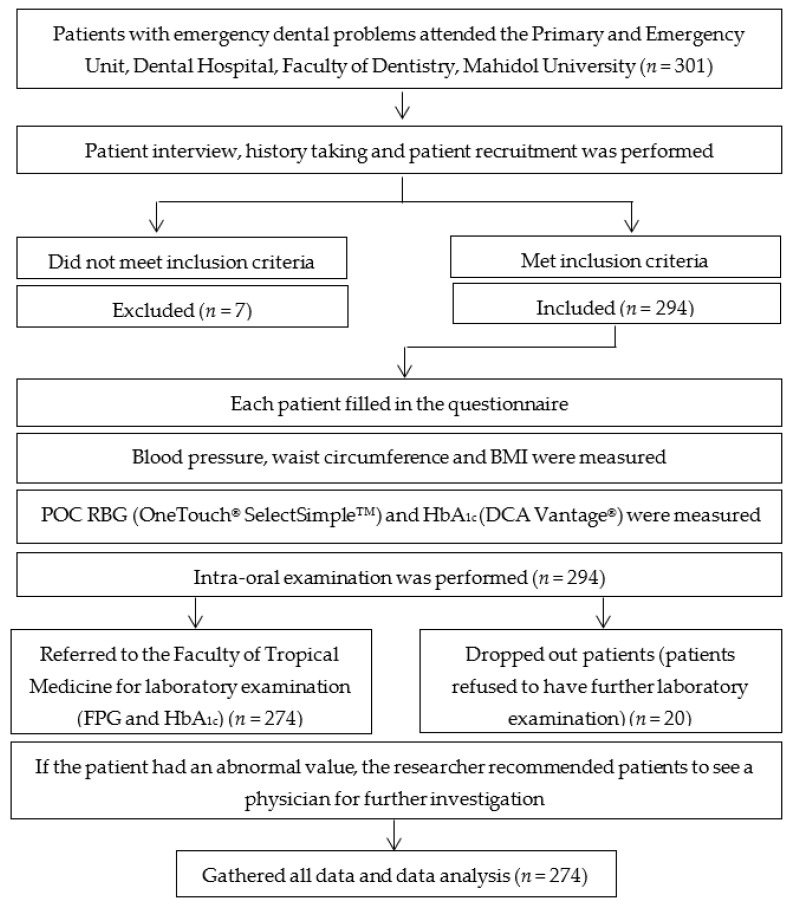
The flow of the research.

This clinical observational study was reviewed and approved by the Institutional Review Board, Faculty of Dentistry/Faculty of Pharmacy, Mahidol University (MU-DT/PY-IRB 2013/010.1902). All subjects gave written informed consent prior to participation in the study. Inclusion criteria were patients aged between 20–70 years old who had no history of type 2 DM, needed emergency dental care, and attended the Primary and Emergency Unit of the Faculty of Dentistry, Mahidol University, Bangkok, Thailand. These consecutive patients that met the inclusion criteria were selected and required to fill in the demographic investigation form and a questionnaire. Exclusion criteria were severe anemia, polycythemia, secondary DM, pregnancy, or taking steroids, glucose-lowering medication, or chemotherapy. Patients who could not answer the questionnaire were also excluded. The flow of this study is presented in Figure 1.

Sample size calculation was determined, using the estimate prevalence of hyperglycemia detected by RBG and HbA_1c_ and a formula for comparative two proportion [15]. At a significant level of 95%, power of 80%, estimated occurrence of hyperglycemia in dental setting being 30% [12] and hypothesized difference in prevalence of hyperglycemia between the two tests at 17%, the minimum computed sample size was 238. This was adjusted to 274 respondents per group.

### 2.2. Demographic Data Collection

Demographic data collection was performed according to our previous study [12]. Briefly, a structured questionnaire was used to collect data regarding the patient’s sex, age, marital status, type of work, smoking, alcohol consumption, and history of medical illness. Risk factors and symptoms of DM were also interviewed as the second part of the questionnaire. Symptoms of DM including polyuria, polydipsia, polyphagia, weight loss, blurred vision, paresthesia, taste disturbance, stress, bad breath, halitosis, and insomnia were also retrieved from each subject. Systolic and diastolic blood pressures were measured from the right arm in the seated position after the subject rested for at least 5 min using an automatic sphygmomanometer (Omron HEM-7221^®^, Omron Healthcare Co., Kyoto, Japan). Bodyweight was measured with a mechanical balance to the nearest 1.0 kg. Standing height was measured and the body mass index (BMI) was calculated as weight (kg) divided by height square (m^2^). Overweight was defined as a BMI over 23 kg/m^2^. Waist circumference ≥ 80 cm in females or ≥90 cm in males indicated central obesity [16].

### 2.3. Periodontal Examination

Study subjects received a full-mouth periodontal examination by 2 well-trained and experienced dentists (MS and TT). Periodontal examination was performed at the dental clinic using mouth mirrors and a manual periodontal probe (North Carolina periodontal probe UNC-15 Hu Friedy Manufacturing, Inc., Chicago, IL, USA) with an artificial dental unit light. Probing depth (PD) and recession were measured on all teeth except the third molar in 6 locations. The level of clinical attachment loss (CAL) was calculated from PD and recession, and it was represented as the distance from the cemento-enamel junction to the base of the periodontal pocket. Bleeding on probing (BOP) was recorded dichotomously as either present or absent. BOP was determined to be positive if hemorrhage occurred within 15 s. Each participant was given instructions regarding dental treatment needs.

Subjects’ periodontal status was classified into 3 levels, including (1) severe, (2) moderate, (3) mild or no periodontitis according to the extent and severity of periodontal disease using the following criteria: severe periodontitis (≥2 interproximal sites with CAL ≥ 6 mm (not on the same tooth) and ≥1 interproximal site with PD ≥ 5 mm), moderate periodontitis (≥2 interproximal sites with CAL ≥ 4 mm (not on same tooth) and ≥2 interproxmal site with PD ≥ 5 mm) and mild or no periodontitis (neither severe nor moderate periodontitis). In the questionnaire, the percentage of BOP site, percentage of the site with PD ≥ 5 mm, the number of missing teeth except for the third molar, and the total number of teeth that decayed, missed, and filled (DMF) were recorded.

### 2.4. Glycemic Measurement

For POC measurement, two blood drops were obtained from each participant, and each was placed on a separate applicator. The researcher performed a random capillary blood glucose (RBG) measurement with a portable blood glucose testing system (OneTouch^®^ SelectSimple™ blood glucometer test strips, LifeScan, Johnson and Johnson Inc., Charleston, SC, USA). The other drop of blood was used for the measurement of POC HbA_1c_. The DCA Vantage^®^ analyzer (Siemens Medical Solutions Diagnostics, Tarrytown, NY, USA), that quantitatively measured the percentage of HbA_1c_ in blood, based on latex agglutination inhibition immunoassay methodology, was used.

After initial POC RBG and POC HbA_1c_ were measured, all patients who had undergone POC testing were requested to come back on a convenient day (within 1 week) for a confirmation of their glycemic conditions using hospital-based laboratory methods including FPG and HbA_1c_ at the Faculty of Tropical Medicine, Mahidol University.

- Hyperglycemia.

Hyperglycemia in this study was defined according to the POC testing, including RBG and POC HbA_1c_. We used 2 different cut-points of RBG at 110 and 140 mg/dl to indicate hyperglycemia, as previously reported in 2 separate studies, respectively [17,18]. POC hyperglycemia was also defined as a HbA_1c_ ≥ 5.7% and this level was found to be very sensitive in identifying people with potential hyperglycemia [1].

- Potential type 2 DM.

Random blood glucose (RBG) test was also used to screen potential DM when DM symptoms are present along with the RBG level of 200 mg/dl or higher [1].

- AGR.

According to the ADA, an FPG level of less than 100 mg/dl was classified as normal, between 100–125 mg/dl was classified as prediabetes and more than or equal to 126 mg/dl was classified as DM [1]. In addition, for HbA_1c_, a level of less than 5.7% was considered to be normal, a HbA_1c_ between 5.7% and 6.4% was considered to be prediabetes, and a HbA_1c_ level greater than or equal to 6.5% was considered to be DM [1,4]. In our study, subjects with FPG ≥ 100 mg/dl or HbA_1c_ ≥ 5.7% were classified as having AGR. Therefore, AGR in this study included both prediabetes and potential type 2 DM.

To convert the plasma glucose level from mg/dl to mmol/L, the value of mg/dl should be divided by 18. For example, plasma glucose level of 110 mg/dl equals 110/18 or 6.1 mmol/L.

### 2.5. Statistical Analysis

All analyses were completed using SPSS, version 22.0 statistical software (SPSS Inc., Chicago, IL, USA). Sensitivity, specificity, positive predictive value (PPV) and negative predictive value (NPV) were compared between methods. Receiver operating characteristic (ROC) analysis was performed using hospital-based laboratory FPG and HbA_1c_ as gold standards for the diagnosis of AGR and potential type 2 DM.

## 3. Results

### 3.1. Subjects’ Characteristics

A total of 274 consecutive subjects who attended the Primary and Emergency Unit of the Faculty of Dentistry, Mahidol University, with complete data, were included in this study. Characteristics of subjects are presented in Table 1. Overall, 39% were male, and 61% were female. The mean age was 43 ± 15 years old. Approximately half of the subjects were younger than 40 years old (49%), single, and had a BMI ≥ 23 kg/m^2^. Over 60% of male and approximately half of the female subjects had central obesity. Most subjects were non-smokers, had no symptoms of type 2 DM, and had no family history of DM. Approximately 25% of subjects had high blood pressure, and 19% of subjects had severe periodontitis (Table 1).

### 3.2. Prevalence of Hyperglycemia and Potential Type 2 DM

The prevalence of hyperglycemia defined as POC HbA_1c_ ≥ 5.7% was 49%. After the confirmation using the laboratory-based FPG and HbA_1c_, the prevalence of AGR was 25% and 17%, respectively (Table 2). Regarding RBG, 37%, 56% and 7% had RBG <110 mg/dl, between 110–200 mg/dl and >200 mg/dl, respectively. After evaluation with FPG by the laboratory, 75%, 20% and 4% had FPG <100 mg/dl, between 100–125 mg/dl and ≥126 mg/dl, respectively. When the laboratory HbA_1c_ was performed, 83%, 13% and 4% had HbA_1c_ <5.7%, between 5.7–6.4% and ≥6.5%, respectively (Table 2). Since another study suggested RBG level ≥ 140 mg/dl to increase the specificity of the screening for hyperglycemia in dental patients [18], the prevalence of hyperglycemia defined as RBG ≥ 140 mg/dl was also evaluated in this study. As shown in Table 2, the prevalence of hyperglycemia was reduced to 32%.

### 3.3. Agreement between Hospital-Based Laboratory Measurement and POC Testing

We next investigated the sensitivity, specificity, positive predictive values (PPV), and negative-predictive values (NPV) of the POC testing for AGR and potential type 2 DM using hospital-based laboratory measurements as gold standards (Table 3 and Table 4). In Table 3, the hospital-based laboratory FPG levels of ≥100 mg/dl and ≥126 mg/dl were used as cut-off levels for AGR and potential type 2 DM, respectively. In Table 4, the hospital-based laboratory HbA_1c_ levels of ≥5.7% and ≥6.5% were used as cut-off levels for AGR and potential type 2 DM, respectively.

Initially, the validity of the test for identifying AGR was performed using ROC analysis (Table 3 and Table 4). First, the AUC was calculated using laboratory FPG ≥ 100 mg/dl as a diagnosis of AGR (Table 3). According to the evaluation, the performance of RBG cut-off point of 110 mg/dl, RBG cut-off point of 140 mg/dl and POC HbA_1c_ in predicting FPG-defined AGR was moderate (AUC = 0.65; 95% CI 0.59–0.70, 0.69; 95% CI 0.62–0.75 and 0.72; 95% CI 0.67–0.78, respectively).

Next, ROC analysis to identify subjects with potential type 2 DM was performed using FPG ≥ 126 mg/dl as a gold standard for the diagnosis of potential type 2 DM (Table 3). Based on the ROC curves, the performances of RBG ≥ 200 mg/dl with symptoms of DM and POC HbA_1c_ in predicting FPG-defined type 2 DM was high (AUC = 0.94; 95% CI 0.86–1.0 and 0.99; 95% CI 0.98–0.99, respectively).

Subsequently, the validity of the test for identifying AGR was performed using ROC analysis and laboratory-based HbA_1c_ ≥ 5.7% as a diagnosis of AGR (Table 4). The performances of RBG cut-off point of 110 mg/dl, RBG cut-off point of 140 mg/dl, and POC HbA_1c_ in predicting laboratory HbA_1c_-defined AGR were also moderate and comparable to that using laboratory FPG ≥ 100 mg/dl as a diagnosis of AGR (AUC = 0.63; 95% CI 0.57–0.69, 0.69; 95% CI 0.62–0.77 and 0.81; 95% CI 0.78–0.84, respectively).

Finally, the ROC analysis was performed using laboratory-based HbA_1c_ ≥ 6.5% as a gold standard for the diagnosis of DM (Table 4). Based on the ROC curves the performances of RBG ≥ 200 mg/dl with symptoms of DM and POC HbA_1c_ ≥ 6.5% in predicting HbA_1c_-defined potential DM was high (AUC = 0.90; 95% CI 0.79–1.00 and 0.99; 95% CI 0.98–0.99, respectively).

## 4. Discussion

In our previous study, dental patients were screened for potential hyperglycemia using POC HbA_1c_ and we found that the prevalence of hyperglycemia defined as POC HbA_1c_ ≥ 5.7% was 33.8% [12]. In this study, we re-investigated the prevalence of hyperglycemia and confirmed the prevalence of AGR using hospital-based laboratory methods. It was found that 49% of subjects had hyperglycemia defined as POC HbA_1c_ ≥ 5.7% (Table 2). In addition, the prevalence of subjects with RBG ≥ 110 mg/dl was as high as 63%. The prevalence was reduced to 32% when the cut-off level was increased to ≥140 mg/dl and this prevalence was comparable to a study by Jadhav and colleagues who reported the prevalence of hyperglycemia in dental patients to be 35% using this RBG cut-off level of 140 mg/dl [18]. The high prevalence in our current study may be due to the fact that the subjects in this study were dental patients who had emergency dental problems and requested emergency dental treatment. It is well established that patients with type 2 DM or prediabetes have a higher risk of periodontal disease and other dental problems [19,20]. This might have some influence in causing this high prevalence and implied that a significant portion of patients with dental problems might have undiagnosed hyperglycemia.

When the hospital-based laboratory methods were utilized to reevaluate the prevalence of AGR and potential type 2 DM, we found that the prevalence of AGR was 25% and 17% when FPG ≥ 100 mg/dl and laboratory-based HbA_1c_ ≥ 5.7% were used, respectively (Table 2). The prevalence of 25% and 17% were higher to the estimated prevalence of hyperglycemia in the Thai population (13%) [14]. When the prevalence of potential type 2 DM was considered, this prevalence was 4% in both measurements using the cut-off levels of FPG ≥ 126 mg/dl and HbA_1c_ ≥ 6.5%. This prevalence was higher than that reported in the Thai population by Aekplakorn and colleagues as well (2.3% defined as FPG ≥ 126 mg/dl). As stated before, the subjects who came to receive dental treatment might have an underlying hyperglycemic condition. Therefore, screening of hyperglycemia in a dental setting using POC methods is worth conducting, and we may be able to detect a significant portion of patients with undiagnosed type 2 DM.

To investigate the performance of POC HbA_1c_ in identifying subjects with AGR, the sensitivities and specificities between the POC HbA_1c_ and hospital-based laboratory methods, including FPG and laboratory-based HbA_1c_ were analyzed. The sensitivity was 82%, and the specificity was 62% when the POC HbA_1c_ ≥ 5.7% was compared to FPG ≥ 100 mg/dl (Table 3). The sensitivity increased to 100% when POC HbA_1c_ ≥ 5.7% was compared to laboratory-based HbA_1c_ ≥ 5.7% (Table 4) but the specificity was similar to that compared to laboratory FPG (62%). In terms of screening for potential type 2 DM, the sensitivity and specificity were 100% and 97% compared to both measurements using FPG ≥ 126 mg/dl and HbA_1c_ ≥ 6.5% (Table 3 and Table 4). This result indicates that screening for AGR and potential type 2 DM using POC HbA_1c_ is possible, with moderate to high sensitivity and specificity compared to the standardized methods, and this screening method should be encouraged in the dental setting.

Comparing our result to a previous study by Genco and colleagues conducted in the U.S.A., 1022 dental patients were screened for hyperglycemia. Of these, 416 (41%) had HbA_1c_ ≥ 5.7% and were referred to see their physicians and 35% of these subjects received a final diagnosis from their physicians within 1 year. The diagnoses were type 2 DM (12%), high risk of developing type 2 DM (23%), and no type 2 DM (64%) [21]. Recently in 2019, changes in screening practices for prediabetes and type 2 DM since the recommendation for HbA_1c_ testing have been reported and encouraged [22]. One study recruited 12,772 eligible patients and reported that when these patients diagnosed with hyperglycemia from the first screening were followed, only 26% of patients screened with blood glucose levels as opposed to 36% of patients screened with HbA_1c_ were diagnosed to have continuous hyperglycemia. Hence this result encouraged the use of POC HbA_1c_ as a screening tool for referral or follow-up of patients with hyperglycemia.

The reason that we preferred RBG was because of the availability for a dental professional to possess a glucometer. According to Table 3 and Table 4, the RBG with different cut-off levels (≥110 mg/dl or ≥ 140 mg/dl) were used for screening of hyperglycemia compared to laboratory FPG and HbA_1c_. The level of RBG ≥ 110 mg/dl was used since it was recommended in a study by Herman et al. that this level of RBG exhibited good sensitivity in identifying people with previously undiagnosed DM regardless of age and time since last food or drink [13]. It was demonstrated that when using the cut-off level of RBG ≥ 110 mg/dl, the sensitivity was high compared to laboratory FPG (85%) (Table 3) and HbA_1c_ (85%) (Table 4), however, the specificity was quite low (approximately 40% compared to both techniques) (Table 3 and Table 4). When the levels of RBG ≥ 140 mg/dl was used, it was obvious that the sensitivities were reduced to 60% and 64% and the specificities were increased to 77% and 74% compared to FPG and laboratory-based HbA_1c_, respectively (Table 3 and Table 4). When the levels of RBG were used for the screening of potential type 2 DM (POC RBG ≥ 200 mg/dl), the sensitivities and specificities were very high (more than 80%). A major objective of most screening tests is to reduce morbidity or mortality in the population group being screened for the disease by early detection [23]. Although the result suggested that RBG might be more specific in identifying dental patients with potential type 2 DM, we still believe that the RBG could give strong benefits for the screening of dental patients with AGR. Our result also suggested that the cut-off level of RBG ≥ 110 mg/dl could be used with high sensitivity to be able to screen more patients who might have AGR.

According to Table 3 and Table 4, the performances of RBG and POC HbA_1c_ in predicting FPG-defined AGR were evaluated. Based on the ROC curves, all methods exhibited moderate performance in predicting patients with AGR when FPG was used for the diagnosis. If the laboratory-based HbA_1c_ was to be used as a diagnosis of having AGR, the performances of RBG and POC HbA_1c_ in predicting laboratory-based HbA_1c_-defined AGR were moderate as well. These performances in predicting patients with potential type 2 DM were increased compared to the performances in predicting patients with AGR. This result suggested that using these POC methods in screening dental patients with undiagnosed type 2 DM might exhibit higher validity than in screening patients with AGR.

In a previous population-based survey of 795 people aged 35–60 in rural areas of Uganda, Mayega et al. determined FPG using glucometer and POC HbA_1c_ using A1cNow^®^ kit and evaluated the relationship between FPG-defined DM status and POC HbA_1c_ values [9]. From their study, with FPG as the reference, an agreement between FPG and POC HbA_1c_ in classifying undiagnosed type 2 DM, was moderate (AUC = 0.75). It is possible that the performance in our study was high since our subjects were dental patients and the subjects in the study by Mayega et al. were the general population. Since the method to identify dental patients with hyperglycemia was only retrieving a drop of blood, we assumed that screening of dental patients with hyperglycemia is beneficial thus that early diagnosis of patients with hyperglycemia can be recognized and may result in early referral and prevention of patients from having complications from chronic hyperglycemia.

One of the limitations in this study might be the low number of subjects identified to have undiagnosed type 2 DM. More subjects may be needed in future studies thus that more patients with undiagnosed type 2 DM will be identified. Moreover, we screened subjects with anemia using only the questionnaire and not the laboratory measurement. Since the measurement of POC HbA_1c_ using the DCA Vantage^®^ analyzer can be affected by abnormal red blood cells, undiagnosed anemia might have influenced the prevalence of hyperglycemia in this study. Despite these limitations, we found that POC HbA_1c_ and RBG had the potential ability to identify dental patients with undiagnosed type 2 DM and, to some extent, for AGR. It is well-established that early detection and appropriate metabolic management of affected individuals can significantly delay the development of most complications. This will provide dental professionals with a tool to directly involve themselves in the healthcare of the patients seen in the dental clinic, particularly in the identification of patients with undiagnosed type 2 DM.

## 5. Conclusions

The results from this study indicated that POC testing, including POC HbA_1c_ and RGB, could be used as a potential tool for screening of subjects with potential type 2 DM and AGR in a dental setting.

## Figures and Tables

**Table 1:** Subjects’ characteristics distributed according to laboratory measurements.

Characteristics	Total	Laboratory HbA_1c_		FPG	
	*n* (%)	<5.7% *n* (%)	≥5.7% *n* (%)	<100 mg/dl *n* (%)	≥100 mg/dl *n*(%)
Gender					
Male	106 (38.7)	80 (75.5)	26 (24.5)	71 (67.0)	35 (33.0)
Female	168 (61.3)	147 (87.5)	21 (12.5)	135 (80.4)	33 (19.6)
Age					
≤40 y	134 (48.9)	115 (85.8)	19 (14.2)	113 (84.3)	21 (15.7)
41–60 y	90 (32.9)	74 (82.2)	16 (17.8)	63 (70.0)	27 (30.0)
≥61 y	50 (18.3)	38 (76.0)	12 (24.0)	30 (60.0)	20 (40.0)
Marital status					
Single	158 (57.7)	133 (84.2)	25 (15.8)	114 (72.2)	44 (27.8)
Married	77 (28.1)	63 (81.8)	14 (18.2)	60 (77.9)	17 (22.1)
Divorced/widow	39 (14.2)	31 (79.5)	8 (20.5)	32 (82.1)	7 (17.9)
Smoking status					
Non-smoking	203 (74.1)	175 (86.2)	28 (13.8)	158 (77.8)	45 (22.2)
Former smoking	61 (22.3)	45 (73.8)	16 (26.2)	41 (67.2)	20 (32.8)
Current smoking	10 (3.7)	7 (70.0)	3 (30.0)	7 (70.0)	3 (30.0)
Alcohol consumption					
Never	163 (59.5	139 (85.3)	24 (14.7)	124 (76.1)	39 (23.9)
Sometimes	56 (20.4)	42 (75.0)	14 (25.0)	41 (73.2)	15 (26.8)
Usually	55 (20.1)	46 (83.6)	9 (16.4)	41 (74.5)	14 (25.5)
Underlying disease					
Yes	193 (70.4)	166 (86.0)	27 (14.0)	149 (72.2)	44 (22.8)
No	81 (29.6)	61 (75.3)	20 (24.7)	57 (70.4)	24 (29.6)
BMI					
≥23 kg/m^2^	152 (55.5)	118 (77.6)	34 (22.4)	117 (77.0)	35 (23.0)
<23 kg/m^2^	122 (44.5)	109 (89.3)	13 (10.7)	89 (73.0)	33 (27.0)
Waist circumference					
Male					
≥90 cm	70 (66.0)	51 (72.9)	19 (27.1)	46 (65.7)	24 (34.3)
<90 cm	36 (34.0)	29 (80.6)	7 (19.4)	25 (69.4)	11 (30.6)
Female					
≥80 cm	81 (48.2)	64 (79.0)	17 (21.0)	61 (75.3)	20 (24.7)
<80 cm	87 (51.8)	83 (95.4)	4 (4.6)	74 (85.1)	13 (14.9)
Family history of DM					
Positive	84 (30.7)	63 (75.0)	21 (25.0)	63 (75.0)	21 (25.0)
Negative	190 (69.3)	164 (86.3)	26 (13.7)	143 (75.3)	47 (24.7)
Symptoms of DM					
With at least 1 symptom	44 (16.1)	33 (75.0)	11 (25.0)	31 (70.5)	13 (29.5)
Without any symptoms	230 (83.9)	194 (84.3)	36 (15.7)	175 (76.1)	55 (23.9)
Hypertension					
≥140/90 mmHg	66 (24.1)	47 (71.2)	19 (28.8)	44 (66.7)	22 (33.3)
<140/90 mmHg	208 (75.9)	180 (86.5)	28 (13.5)	162 (77.9)	46 (22.1)
Periodontal status					
Mild/none	173 (63.1)	156 (90.2)	17 (9.8)	149 (86.1)	24 (13.9)
Moderate	48 (17.5)	36 (75.0)	12 (25.0)	30 (62.5)	18 (37.5)
Severe	53 (19.3)	35 (66.0)	18 (34.0)	27 (50.9)	26 (49.1)

FPG: fasting plasma glucose.

**Table 2:** Prevalence of hyperglycemia (POC HbA_1c_ ≥ 5.7%, POC RBG ≥ 110 and ≥140 mg/dl), prediabetes and potential type 2 DM according to various methods of detection.

Method	Range	Frequency	Percent
POC HbA_1c_	<5.7%	140	51
	5.7–6.4%	115	42
	≥6.5%	19	7
	≥5.7%	134	49
RBG	<110 mg/dl	101	37
	110–200 mg/dl	154	56
	>200 mg/dl	19	7
	≥110 mg/dl	173	63
RBG	<140 mg/dl	186	68
	140–200 mg/dl	69	25
	>200 mg/dl	19	7
	≥140 mg/dl	88	32
FPG	<100 mg/dl	206	75
	100–125 mg/dl	56	20
	≥126 mg/dl	12	4
	≥100 mg/dl	68	25
Laboratory-HbA_1c_	<5.7%	227	83
	5.7–6.4%	35	13
	≥6.5%	12	4
	≥5.7%	47	17

POC: point-of-care; RBG: random blood glucose.

**Table 3:** Sensitivity, specificity, PPV, and NPV of POC testing compared to hospital-based laboratory testing using FPG levels. (*n* = 274).

Testing	Hospital-Based FPG Level			Sens **	Spec **	PPV	NPV	AUC	95%CI
	Normal * n (%)	AGR * *n* (%)	Total *n* (%)						
**POC HbA_1c_**									
<5.7%	128 (46.72)	12 (4.38)	140 (51.09)	82.35	62.14	41.79	91.43	0.72	0.67–0.78
≥5.7%	78 (28.47)	56 (20.44)	134 (48.91)						
Total	206 (75.19)	68 (24.82)							
**RBG cut-off = 110 mg/dl**									
<110 mg/dl	91 (33.21)	10 (3.65)	101 (36.86)	85.29	44.17	33.53	90.10	0.65	0.59–0.70
≥110 mg/dl	115 (41.97)	58 (21.17)	173 (63.14)						
Total	206 (75.19)	68 (24.82)							
**RBG cut-off = 140 mg/dl**									
<140 mg/dl	159 (58.03)	27 (9.85)	186 (67.88)	60.29	77.18	46.59	85.48	0.69	0.62–0.75
≥140 mg/dl	47 (17.15)	41 (14.96)	88 (32.12)						
Total	206 (75.19)	68 (24.82)							
	Non-DM *	Potential DM *							
**POC HbA_1c_**									
<6.5%	255 (93.07)	0 (0)	255 (93.07)	100.00	97.33	63.16	100	0.99	0.98–0.99
≥6.5%	7 (2.55)	12 (4.38)	19 (6.93)						
Total	262 (95.62)	12 (4.38)							
**RBG**									
<200 mg/dl	254 (92.70)	1 (0.36)	255 (93.07)	91.67	96.95	57.89	99.61	0.94	0.86–1.00
≥200 mg/dl	8 (2.92)	11 (4.02)	19 (6.93)						
Total	255 (93.07)	12 (4.38)							

* Normal indicates FPG levels < 100 mg/dl; AGR indicates prediabetes and potential DM with FPG levels ≥ 100 mg/dl; Non-DM indicates FPG levels < 126 mg/dl; Potential DM indicates FPG levels ≥ 126 mg/dl. ** Sens: sensitivity; Spec: Specificity. PPV: positive predictive value; NPV: negative predictive value; AUC: area under the curve; DM: diabetes mellitus.

**Table 4:** Sensitivity, specificity, PPV, and NPV of POC testing compared to hospital-based laboratory testing using HbA_1c_ levels. (*n* = 274).

Testing	Hospital-Based HbA_1c_ Level			Sens **	Spec **	PPV	NPV	AUC	95%CI
	Normal * n (%)	AGR * *n* (%)	Total *n* (%)						
**POC HbA_1c_**									
<5.7%	140 (51.09)	0 (0)	140 (51.09)	100	61.67	35.07	100	0.81	0.78–0.84
≥5.7%	87 (31.75)	47 (17.15)	134 (48.91)						
Total	227 (82.84)	47 (17.15)							
**RBG cut-off = 110 mg/dl**									
<110 mg/dl	94 (34.31)	7 (2.55)	101 (36.86)	85.11	41.41	23.12	93.07	0.63	0.57–0.69
≥110 mg/dl	133 (48.54)	40 (14.60)	173 (63.14)						
Total	227 (82.84)	47 (17.15)							
**RBG cut-off = 140 mg/dl**									
<140 mg/dl	169 (61.68)	17 (6.20)	186 (67.88)	63.83	74.45	34.09	90.86	0.69	0.62–0.77
≥140 mg/dl	58 (21.17)	30 (10.95)	88 (32.12)						
Total	227 (82.84)	47 (17.15)							
	Non-DM *	Potential DM *							
**POC HbA_1c_**									
<6.5%	255 (93.07)	0 (0)	255 (93.07)	100	97.33	63.16	100	0.99	0.98–0.99
≥6.5%	7 (2.55)	12 (4.38)	19 (6.93)						
Total	262 (95.62)	12 (4.38)							
**RBG**									
<200 mg/dl	253 (92.34)	2 (0.73)	255 (93.07)	83.33	96.56	52.63	99.22	0.90	0.79–1.00
≥200 mg/dl	9 (3.28)	10 (3.65)	19 (6.93)						
Total	262 (95.62)	12 (4.38)							

* Normal indicates HbA_1c_ levels < 5.7%; AGR indicates prediabetes and potential DM with HbA_1c_ levels ≥ 5.7%; Non-DM indicates HbA_1c_ levels < 6.5%; Potential DM indicates HbA_1c_ levels ≥ 6.5%. ** Sens: sensitivity; Spec: Specificity.

## Data Availability

The data presented in this study are available on request from the corresponding author. The data are not publicly available due to ethical issue.
