# Erratum: Suwattipong et al. Comparison of Point-of-Care Testing and Hospital-Based Methods in Screening for Potential Type 2 Diabetes Mellitus and Abnormal Glucose Regulation in a Dental Setting. *Int. J. Environ. Res. Public Health* 2021, *18*, 6459

**DOI:** 10.3390/ijerph182111462

**Published:** 2021-10-31

**Authors:** Muneedej Suwattipong, Thitima Thuramonwong, Chanita Tantipoj, Pornpoj Fuangtharnthip, Supanee Thanakun, Weerapan Khovidhunkit, Siribang-on Piboonniyom Khovidhunkit

**Affiliations:** 1 Dental Hospital, Faculty of Dentistry, Mahidol University, Bangkok 10400, Thailand; muneedej.suw@mahidol.edu (M.S.); katae_katier@hotmail.com (T.T.); 2 Department of Advanced General Dentistry, Faculty of Dentistry, Mahidol University, Bangkok 10400, Thailand; ctantipoj@gmail.com (C.T.); pornpoj.fun@mahidol.ac.th (P.F.); 3 College of Dental Medicine, Rangsit University, Muang Pathum Thani 12000, Thailand; supanee.tha2@gmail.com; 4 Department of Medicine, Faculty of Medicine, Chulalongkorn University, Bangkok 10330, Thailand; wkhovid@gmail.com

## Error in Ethical Approval Number

In the original article [1], it was documented that this clinical observational study was reviewed and approved by the Institutional Review Board, Faculty of Dentistry/Faculty of Pharmacy, Mahidol University (MU-DT/PY-IRB 2013/010.1902). In fact, the ethical approval number should be MU-DT/PY IRB 2017/047.2308.

The authors apologize for this error and would like to reiterate that the scientific conclusions are unaffected.
